# Electro‐clinical features of Mowat–Wilson syndrome: A retrospective study of 31 children in mainland China

**DOI:** 10.1002/epd2.70149

**Published:** 2025-12-27

**Authors:** Yi Ju, Tao‐yun Ji

**Affiliations:** ^1^ Department of Pediatrics Peking University First Hospital Beijing China

**Keywords:** anti‐seizure medications, epilepsy, Mowat–Wilson syndrome, *ZEB2*

## Abstract

**Objective:**

To summarize the electro‐clinical and genetic characteristics of children with Mowat–Wilson syndrome (MWS).

**Methods:**

This study is a hospital‐based case series analyzing clinical data from 31 pediatric patients with MWS and epilepsy treated at Peking University First Hospital between June 2020 and December 2024. Information on seizures, electroencephalographic features, genetic characteristics, treatment, and prognosis was summarized and analyzed using descriptive statistics.

**Results:**

Among the 31 children (16 males and 15 females), seizure onset occurred at a median age of 25.5 months (range: 1–113 months). Eighteen cases (58.1%, 18/31) began with fever‐induced seizures; all 31 children experienced focal seizures, and 16 (51.6%, 16/31) exhibited atypical seizure presentations. Twelve (38.7%, 12/31) experienced seizures accompanied by gastrointestinal (GI) symptoms. Two children had myoclonic seizures, one had epileptic spasms, and another had atypical absence seizures. Ten (32.3%, 10/31) experienced convulsive status epilepticus. Electroencephalographic findings evolved from posterior head–dominant discharges to multifocal or anterior head–dominant discharges, with a significant increase in discharges during sleep. All 31 children had de novo *ZEB2* variants, including 27 with single‐nucleotide variants (SNVs) or insertions/deletions (indels) and four with copy number variants. Among the SNVs/indels, nonsense (13) and frameshift (12) variants predominated. One patient with rare seizures did not receive anti‐seizure medication (ASM). Thirty received ASMs; both levetiracetam and valproic acid, used as monotherapy or in combination, proved effective. Sixteen children achieved seizure control for more than 6 months, and seven maintained seizure control for over 1 year.

**Significance:**

Our findings reveal the electro‐clinical characteristics, genetic variants, and effective treatments associated with MWS, providing an important basis for clinical diagnosis and management.

**Key points**

- We performed a case series analysis to summarize the electro‐clinical and genetic characteristics of children with MWS.
- MWS‐related seizures often begin as fever‐induced events, with focal seizures being the most prevalent; however, nearly half present atypically and may be overlooked.
- Convulsive status epilepticus occurs in approximately one‐third of affected children, and EEG patterns reveal age‐dependent variation.
- *ZEB2* variants are frequent, with a high incidence of de novo nonsense and frameshift mutations.
- LEV and VPA are effective in managing MWS‐related seizures and may serve as first‐line ASMs in affected children.

## 1 INTRODUCTION

Mowat–Wilson syndrome (MWS) (Online Mendelian Inheritance in Man [OMIM]; 235730) is an autosomal dominant disorder caused by variants in the zinc finger E‐box binding homeobox 2 gene (*ZEB2*). It is characterized by marked phenotypic and genetic heterogeneity, with an estimated incidence of one in 50 000 to one in 70 000 individuals.^1^ The syndrome presents with distinctive facial features, such as a bird‐like appearance with a protruding nasal columella, microcephaly, hypertelorism, and prominent earlobes, along with epileptic seizures, developmental delays, hypoplasia or agenesis of the corpus callosum, and multiple congenital anomalies (including Hirschsprung disease [HSCR] and congenital heart defects).

Epilepsy occurs in a high proportion of patients with MWS and significantly affects their quality of life. However, only a few studies have systematically described the electro‐clinical and genetic characteristics associated with epilepsy in MWS.^2^, ^3^, ^4^

Here, we summarize the clinical, particularly seizure‐related, and genetic characteristics of 31 children with MWS who were treated at our hospital, with the aim of improving clinicians' understanding of the disorder and sharing our experience in the comprehensive management of children with MWS and epilepsy.

## 2 MATERIALS AND METHODS

### 2.1 Participants

A total of 31 children with MWS, diagnosed based on typical clinical manifestations and genetic testing, were included in this study. All patients were treated at the Peking University First Hospital between June 2020 and December 2024. The study protocol was approved by the Ethics Committee of Peking University First Hospital (approval no.: 2005–04) and conducted in accordance with the Declaration of Helsinki. Written informed consent was obtained from all participants or their legal guardians.

### 2.2 Methods

#### 2.2.1 Case data collection

Case data were collected and updated through reviews of medical records, outpatient visits, telephone interviews, and online follow‐ups. Follow‐up assessments were conducted every 6 months, with the final evaluation completed in December 2024.

The collected data included as follows:

1. Basic demographic information (name, sex, and date of birth).
2. Epilepsy‐related information, including age at seizure onset, seizure type and frequency, history of convulsive status epilepticus, treatment, and response to anti‐seizure medications (ASMs).
3. Findings from auxiliary examinations, such as electroencephalography (EEG).
4. Other clinical features, including characteristic facial appearance, language, motor and cognitive development, congenital heart disease, constipation, and Hirschsprung disease (HSCR).

#### 2.2.2 Gene variant detection

Whole‐exome sequencing (WES) and copy number variation (CNV) analysis were performed to identify variants in *ZEB2*. To confirm the origin of the variants, Sanger sequencing and quantitative real‐time polymerase chain reaction (qPCR) were used for validation in affected children and their parents. Sequence variants were evaluated based on pathogenicity evidence from at least five in silico prediction tools (SIFT, MutationTaster, PROVEAN, PolyPhen‐2, and CADD) and allele frequencies in the general population. The variants were then interpreted according to the American College of Medical Genetics and Genomics (ACMG) recommendations for pathogenicity determination.^5^, ^6^

#### 2.2.3 Seizure classification

Two epileptologists independently classified seizure types according to the 2017 International League Against Epilepsy (ILAE) criteria,^7^ using both video EEG recordings and clinical semiology.

#### 2.2.4 Grouping

Patients were stratified into two groups according to genetic variant type:

- single‐nucleotide variants (SNVs) or insertion‐deletions (indels) group, and
- CNV group.

### 2.3 Statistical analysis

Descriptive statistics, including frequencies and percentages, were calculated using Microsoft Excel 2021. Categorical variables were expressed as counts (*n*, %) and continuous variables were expressed as median values (months).

## 3 RESULTS

### 3.1 Demographic characteristics

At the final follow‐up, the ages of the children ranged from 2 years 3 months to 12 years 8 months. All 31 children completed the follow‐up period, and one child died of unknown causes. The longest follow‐up duration was 4.5 years.

### 3.2 Clinical characteristics

#### 3.2.1 Seizure characteristics

Seizure onset occurred at a median age of 25.5 months (range: 1–113 months). In 18 cases (58.1%), the first seizure was fever‐triggered. Focal seizures were the most common type (100%, 31/31), but they were often atypical and easily overlooked.

In this study, focal seizures typically presented as hyporesponsiveness (16 cases), motionless staring or gaze deviation, oral automatisms, or mild eyelid blinking. Tonic or clonic manifestations were generally absent.

Twelve patients (38.7%, 12/31) experienced episodes accompanied by gastrointestinal (GI) symptoms, including bloating or constipation before seizures and nausea or vomiting during episodes. Ten patients (32.3%, 10/31) experienced convulsive status epilepticus during the disease course.

Case 3 (SNV/indel group) presented with epileptic spasms at 7 months of age, characterized by eye rolling and upper limb elevation. Case 5 (SNV/indel group) experienced a myoclonic seizure at 3 years and 8 months, with shaking of the right limb, followed by atypical absence seizures at 7 years and 4 months, characterized by reduced eyelid movement and blinking. Case 3 (CNV group) exhibited frequent myoclonic seizures beginning at 2 years and 9 months of age.

#### 3.2.2 Development

All 31 children exhibited developmental delays, most with moderate‐to‐severe impairment.

Regarding language development, most children produced instinctive vocalizations during infancy (e.g., “a,” “mama,” and “baba”), followed by slow linguistic progression. Among 18 children followed beyond 5 years of age, six achieved purposeful articulation, producing simple words and phrases, such as “fruit,” “don't want,” and “don't eat.” Two children could hum or sing short children's songs and perform basic counting tasks.

Motor milestones were delayed relative to peers. Gross motor skills progressed more rapidly than fine motor skills. Among 18 children followed beyond 5 years of age, 15 achieved independent walking, four could run and jump, and nine could grasp simple objects, though grip stability remained poor.

#### 3.2.3 Other clinical features

All 31 children displayed characteristic MWS facial features. Congenital heart disease was present in 15 (48.4%) children, most commonly patent ductus arteriosus (seven cases), patent foramen ovale (five cases), ventricular septal defect (three cases), and atrial septal defect (three cases). Additional anomalies included pulmonary artery sling, mild tricuspid regurgitation, and persistent left superior vena cava (each in one case).

Constipation of varying severity occurred in 14 (45.2%) cases; pathological examination confirmed Hirschsprung disease in seven. Five children had hypospadias, one had cryptorchidism, and one had syringomyelia.

Six cases had ocular anomalies, including internal strabismus (four cases), esotropia (one case), and mild left ophthalmoplegia with anisometropia combined with right‐eye internal strabismus (one case).

#### 3.2.4 Electroencephalography (EEG) findings

All 31 children underwent at least one video EEG examination, yielding 87 datasets (24 recorded at our hospital). Each EEG included data from both wakefulness and sleep. Abnormal findings were observed in all children.

At seizure onset, EEGs primarily demonstrated background slowing and posterior head–dominant discharges in 20 cases. Over time, background activity improved but remained slower than age‐matched norms. Among 18 children followed to at least age 5 years, occipital rhythms (6–8 Hz) were poorly regulated and amplitude modulated.

Interictal discharges evolved from posterior dominance to multifocal or anterior head–dominant patterns. A significant increase in discharge frequency occurred during sleep.

**FIGURE 1:**
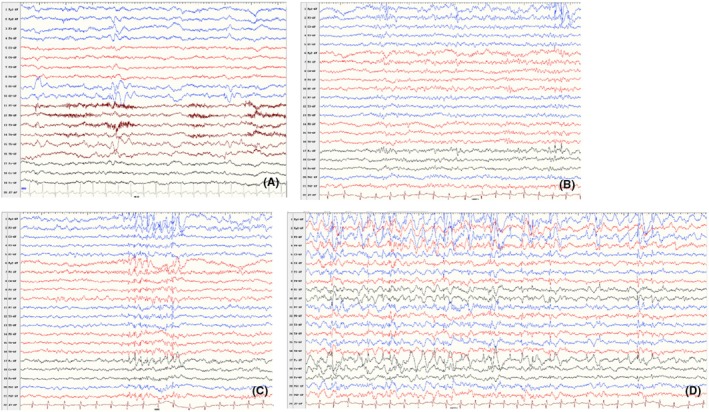
Electroencephalography (EEG) evolution at 1 year 9 months (A) and 5 years 1 month (B–D) in a child with Mowat–Wilson syndrome (MWS) (Case 1, single‐nucleotide variants [SNV]/indel group). (A) Bilateral epileptiform discharges predominantly in the occipital and posterior temporal regions. (B–D) Bilateral anterior cephalic and extensive discharges, with a significant increase in activity during sleep and a discharge index of approximately 60% during sleep. Low‐frequency filter, 1.6 Hz; high‐frequency filter, 70 Hz; paper speed, 30 mm/s; sensitivity, 150 μV/cm.

Electrical status epilepticus during sleep (ESES) was observed in two patients (Cases 1 and 6, SNV/indel group). For Case 1, EEG at 5 years and 1 month revealed a discharge index of approximately 60% during sleep (Figure 1). In Case 6, EEGs at 4 years 4 months and 6 years 3 months revealed a 50% discharge index during sleep.

One patient (Case 3, SNV/indel group) initially presented with epileptic spasms and exhibited hypsarrhythmia. Seizures resolved after treatment, and the final EEG at 4 years 6 months showed left frontal discharges.

Across 10 EEG recordings from 5 patients, clinical seizures, including focal, myoclonic, epileptic spasms, and atypical absence seizures, were recorded.

#### 3.2.5 Neuroimaging

Cranial magnetic resonance imaging (MRI) data were available for 20 of the 31 children. Fourteen (70%) exhibited structural abnormalities, including ventricular dilatation (four cases), corpus callosum dysplasia (three cases), cerebral white matter dysplasia (three cases), delayed myelination (two cases), hippocampal atrophy (one case), and cerebral atrophy (one case).

### 3.3 Genetic testing

Genetic analysis revealed *ZEB2* variants in all 31 children, including 27 SNVs/indels (16 males and 11 females) and 4 CNVs (all females).

Among the SNVs/indels, there were 13 nonsense variants, 12 frameshift variants, and two splice‐site variants. Nineteen variants were located in exon 8, two each in exons 6 and 10 (Cases 13, 17, 23, and 24), one in exon 3 (Case 22), and one in exon 7 (Case 20). Two splice‐site variants were located in intronic regions (c.‐69‐2A>T and c.331+1delG).

**FIGURE 2:**
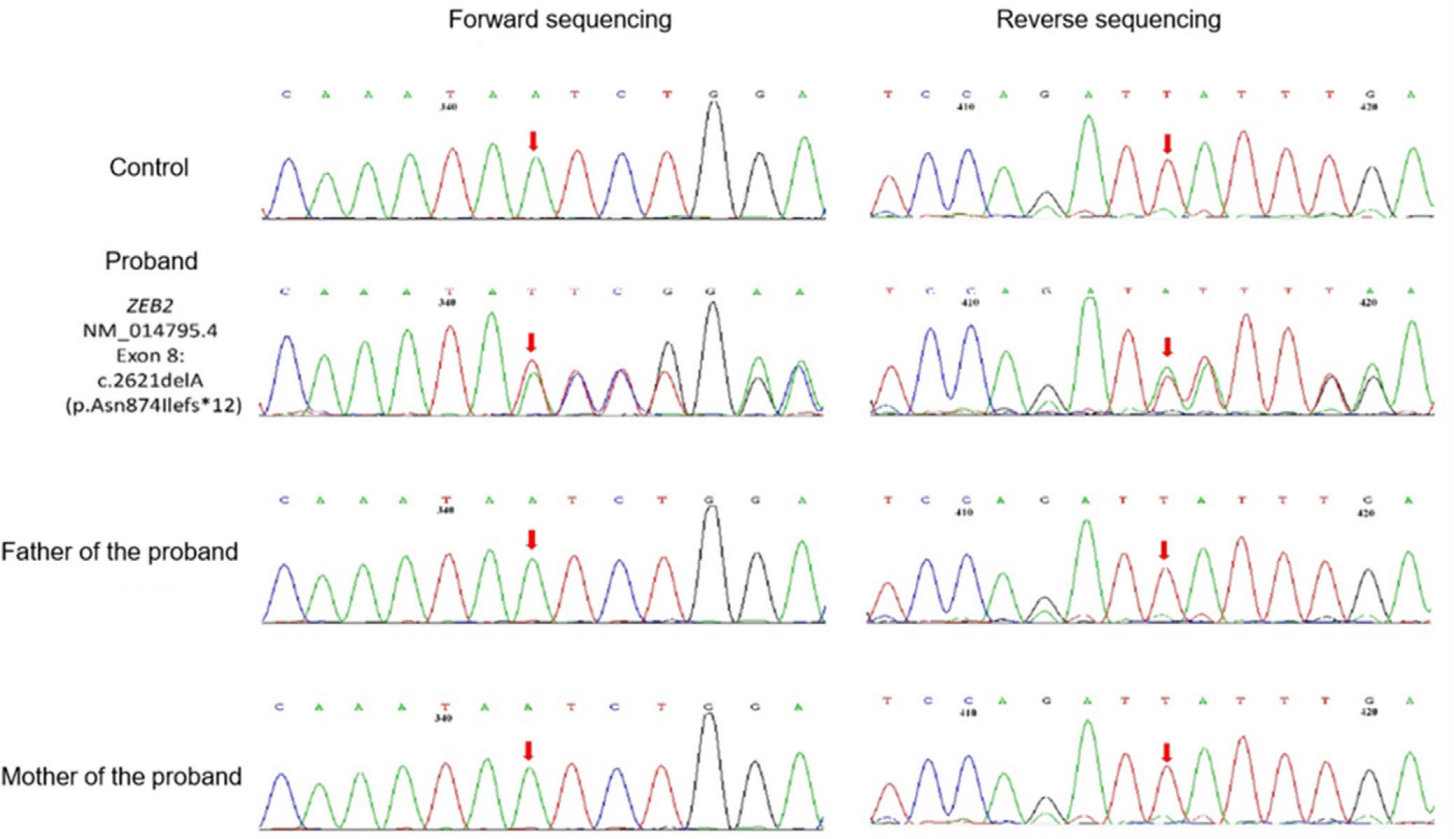
Sanger sequencing of the *ZEB2* gene in the single‐nucleotide variants (SNVs)/indels group (Case 21) and family members. The red arrow indicates a single‐nucleotide deletion (c.2621delA) in exon 8 of *ZEB2*, resulting in a frameshift mutation (p.Asn874Ilefs*12). This change is clearly visible in both the forward and reverse Sanger sequencing traces of the proband and is absent in the chromatograms of both parents, confirming its de novo origin.

All SNVs/indels and CNVs were de novo. Based on the ACMG classification, 20 variants were pathogenic and six were likely pathogenic. Ten SNVs/indels had been previously reported as pathogenic, whereas 17 SNVs/indels and all four CNVs were newly identified in this study and considered novel. The c.2083C>T variant was detected in two children (Cases 11 and 12). The SNV/indel identified in Case 21 is shown in Figure 2.

**FIGURE 3:**
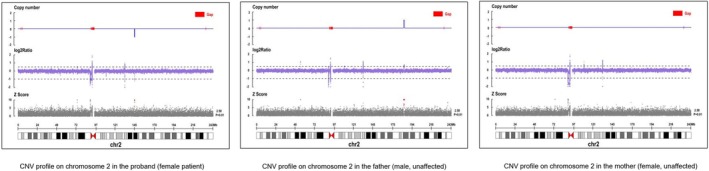
Copy number variation (CNV) profile of Case 1 (CNV group) and family members. The analysis reveals a 136.01 kb de novo heterozygous deletion at position seq[GRCh37]de1(2)(q22.3q22.3) (chr2:145, 148, 150–145, 284, 161). The deletion is present in the proband but absent in both parents, who exhibit wild‐type profiles.

Four CNVs were identified, with deletion sizes of 136.01 kb, 0.35, 12.16, and 6.85 Mb—all de novo. According to ACMG criteria, two were classified as pathogenic, one as likely pathogenic, and one as a variant of uncertain significance. The CNV detected in Case 1 is shown in Figure 3.

**FIGURE 4:**
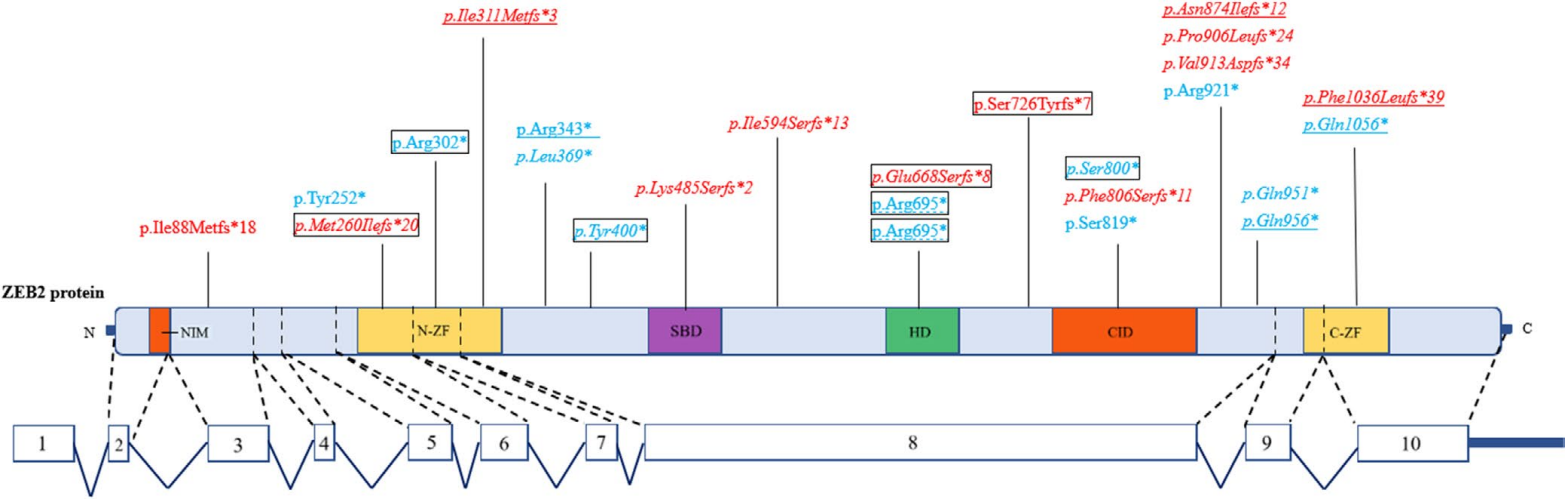
Schematic representation of the ZEB2 protein (NP_014795.4) showing the locations of genetic variants identified in this cohort. Intronic splice‐site variants (c.‐69‐2A>T and c.331+1delG) are not shown because they lie outside the mature protein sequence. The *ZEB2* protein domains and corresponding amino acid positions (in parentheses) are as follows: Minimal nucleosome remodeling and deacetylase interaction motif (NIM), N‐terminal zinc finger cluster (N‐ZF), Smad‐binding domain (SBD), homeodomain (HD), CtBP‐interacting domain (CID), and C‐terminal zinc finger cluster (C‐ZF). Variants are color‐coded by type: Red font, frameshift variants; blue font, nonsense variants. Italics represent newly identified variants. Variants observed in two independent patients are connected by dashed lines. Variants associated with seizure freedom or seizure control ≥1 year are underlined by solid lines, while variants linked with persistent epileptic encephalopathy are framed.

The genetic characteristics of all 31 children are summarized in Tables 1 and 2, and the locations of the identified SNVs/indels within the *ZEB2* gene are shown in Figure 4.

**TABLE 1:** Summary of genetics and seizure characteristics of Mowat–Wilson syndrome (MWS) due to gene point variants.

Patient	Gender/age at last follow‐up	*ZEB2* mutation	Previously reported	Age at onset of seizures	Fever‐triggered seizures	Convulsive status epilepticus	Seizure type and features	EEG		Treatment	
								Background activity	Interictal discharges	ASM(s)	Response
1	F/5y4m	c.2712del p.Pro906Leufs*24	−	14m	+	−	Focal seizure, combined with gastrointestinal symptoms, atypical seizure presentation	Slowing	Bilateral posterior head area → widespread	VPA, LEV	Uncontrolled
2	F/2y9m	c.2738_2742del p.Val913Aspfs*34	−	16m	−	−	Focal seizure, atypical seizure presentation	Slowing	Bilateral anterior head area	LEV	Uncontrolled
3	M/5y9m	c.2456C>G p.Ser819*	+	6m	−	−	Focal seizures combined with gastrointestinal and epileptic spasms	Hypsarrhythmia→Slowing	Bilateral posterior head area → widespread	VPA, TPM, LTG	8 m controlled
4	M/8y2m	c.1027C>T p.Arg343*	+	47m	+	−	Focal seizure, combined with gastrointestinal symptoms, atypical seizure presentation	Slowing→normal	Bilateral posterior head area	LEV	Controlled more than 2y
5	F/8y	c.2761C>T p.Arg921*	+	17m	+	−	Focal seizures, myoclonic seizures, atypical absence seizures, atypical seizure presentation	Slowing	Widespread, and multifocal	ZNS, PER	Uncontrolled
6	M/6y10m	c.1200 T>A p.Tyr400*	−	25m	−	+	Focal seizure, combined with gastrointestinal symptom	Slowing→normal	Widespread and mutifocal, bilateral posterior head area dominant	VPA, PER	9 m controlled
7	M/4y3m	c.2177_2180del p.Ser726Tyrfs*7	+	51m	+	+	Focal seizure	Normal	Bilateral posterior head area	LEV	Uncontrolled
8	F/5y4m	c.2851C>T p.Gln951*	−	64m	+	−	Focal seizure, atypical seizure presentation	Slowing	Right posterior head area → widespread and multifocal	VPA, LEV, LCM	Uncontrolled
9	M/3y8m	c.2866C>T p.Gln956*	−	13m	+	−	Focal seizure, atypical seizure presentation	Slowing	Bilateral anterior head area	LEV	14 m controlled
10	M/5y2m	c.1454del p.Lys485Serfs*2	−	8m	−	−	Focal seizure, combined with gastrointestinal symptoms, atypical seizure presentation	Slowing	Bilateral posterior head area →widespread and multifocal	VPA	Uncontrolled
11	F/5y10m	c.2083C>T p.Arg695*	+	32m	+	+	Focal seizure, combined with gastrointestinal symptoms, atypical seizure presentation	Slowing	Bilateral posterior head area	LEV, ZNS, PER	Uncontrolled
12	F/5y3m	c.2083C>T p.Arg695*	+	13m	+	+	Focal seizure, atypical seizure presentation	Slowing	Bilateral posterior head area → widespread and multifocal	VPA, PER	Uncontrolled
13	F/2y7m	c.3108_3135delinsTTTGTGTTCGTG p.Phe1036Leufs*39	−	12m	+	−	Focal seizure, atypical seizure presentation	Normal	Bilateral posterior head area	LEV	12 m controlled
14	M/11y2m	c.‐69‐2A>T	−	113m	−	−	Focal seizure, combined with gastrointestinal symptom	Normal	Bilateral occipital areas	VPA	21 m controlled
15	M/7y10m	c.933del p.Ile311Metfs*3	−	37m	−	−	Focal seizure	Slowing→normal	Bilateral posterior head area	VPA, LEV	18 m controlled
16	M/12y8m	c.1106_1115delinsAG p.Leu369*	−	13m	+	−	Focal seizure, combined with gastrointestinal symptoms, atypical seizure presentation	Normal	Anterior head area	VPA, OXC, ZNS	10 m controlled
17	F/4y8m	c.779dup p.Met260Ilefs*20	−	27m	Unknown	+	Focal seizure	Normal	Right central region	VPA, LEV, PER	Uncontrolled
18	M/2y3m	c.1779del p.Ile594Serfs*13	+	1m	Unknown	−	Focal seizure	Slowing	The left posterior head area	LEV	8 m controlled
19	M/4y11m	c.2002del p.Glu668Serfs*8	−	Unknown	Unknown	+	Focal seizure, combined with gastrointestinal symptom	Slowing	Widespread and multifocal, bilateral posterior head–dominant	LEV	7 m controlled
20	M/4y8m	c.904C>T p.Arg302*	+	12m	+	+	Focal seizure	Normal	Bilateral occipital areas	VPA, LEV	Uncontrolled
21	F/4y5m	c.2621del p.Asn874Ilefs*12	−	30m	+	−	Focal seizure	Slowing	Anterior head area	−	−
22	M/9y8m	c.264_267del p.Ile88Metfs*18	+	36m	+	−	Focal seizure, combined with gastrointestinal symptoms, atypical seizure presentation	Slowing	Bilateral posterior head area → widespread and multifocal	VPA, OXC	Uncontrolled
23	M/3y4m	c.3166C>T p.Gln1056*	−	15m	−	−	Focal seizure	Normal	Anterior head area	LEV	Controlled more than 2y
24	F/8y9m	c.756C>A p.Tyr252*	+	88m	−	−	Focal seizure	Slowing	Bilateral posterior head area → widespread and multifocal	ZNS	Uncontrolled
25	M/3y1m	c.2399C>A p.Ser800*	−	14m	+	+	Focal seizure	Slowing	Bilateral anterior head area	VPA	6 m controlled
26	M/8y9m	c.331+1del	−	34m	−	−	Focal seizure, atypical seizure presentation	Slowing	Widespread and multifocal	VPA, LEV, LCM	4 m controlled
27	F/4y11m	c.2417del p.Phe806Serfs*11	−	32m	+	−	Focal seizure, combined with gastrointestinal symptoms, atypical seizure presentation	Slowing	Bilateral posterior head area	VPA, LEV	10 m controlled

Abbreviations: ASM(s), anti‐seizure medication(s); EEG, electroencephalogram; F, female; LCM, lacosamide; LEG, lamotrigine; LEV, levetiracetam; M, male; m, month; OXC, oxcarbazepine; PER, perampanel; TPM, topiramate; VPA, valproic acid; y, year; ZNS, zonisamide.

**TABLE 2:** Summary of genetics and seizure characteristics of Mowat–Wilson syndrome (MWS) due to gene copy number variations.

Patient	Gender/age at last follow‐up	Variant location (GRCh37/hg19) and deletion size	Previously reported	Age at onset of seizures	Fever‐triggered seizures	Convulsive status epilepticus	Seizure type and features	EEG		Treatment	
								Background activity	Interictal discharges	ASM(s)	Response
1	F/4y3m	chr2:145148150_145284161 136.01Kb	−	14m	+	+	Focal seizure	Slowing	Bilateral anterior and posterior head area	VPA, CLB, PER	Uncontrolled
2	F/8y1m	chr2:145000000_145351228 0.35 Mb	−	27m	+	−	Focal seizure	Slowing	Multifocal, left posterior head area dominant	VPA, LEV, PER	23 m controlled
3	F/6y3m	chr2:136880000_149040000 12.16 Mb	−	26m	+	+	Focal seizure, myoclonic seizure, combined with gastrointestinal symptoms, atypical seizure presentation	Slowing	Bilateral posterior head area → widespread	VPA, LEV	7 m controlled
4	F/7y4m	chr2:138434153_145285163 6.85 Mb	−	34m	−	−	Focal seizure, atypical seizure presentation	Slowing	Bilateral posterior head area	LEV, LCM	8 m controlled

Abbreviations: ASM(s), anti‐seizure medication(s); CLB, clobazam; EEG, electroencephalogram; F, female; LCM, lacosamide; LEV, levetiracetam; M, male; m, month; PER, perampanel; VPA, valproic acid; y, year.

### 3.4 Seizure treatment and outcomes

Among the 31 children with seizures, one child did not receive ASMs due to infrequent episodes, whereas the remaining 30 children were treated with ASMs and followed up regularly.

ASMs were administered as monotherapy in 12 children at the last follow‐up. Eight received levetiracetam (LEV), three received valproic acid (VPA), and one received zonisamide (ZNS). Eighteen children were treated with combination therapy, including LEV + VPA in five cases, LEV + VPA + perampanel (PER) in seven cases, and other combinations involving ZNS (three cases), lacosamide (two cases), lamotrigine (one case), and topiramate (one case).

#### 3.4.1 Seizure control

At the last follow‐up, two children achieved seizure control for more than 2 years, both on LEV monotherapy.

Five children maintained seizure control for 1–2 years; two treated with LEV monotherapy, one with VPA monotherapy, one with LEV + VPA, and one with LEV + VPA + PER combination therapy.

Nine children achieved seizure control lasting 6–12 months, including two on LEV monotherapy, one on VPA monotherapy, two on LEV + VPA, and four on other combinations involving either LEV or VPA.

One child achieved seizure control for 4 months after the addition of VPA and remained under observation.

## 4 DISCUSSION

Seizures are among the most common neurological manifestations of MWS in children. Cordelli et al.^2^ reported that approximately 70%–75% of children with MWS experience seizures, while Ivanovski et al.,^8^ in a summary of 87 patients (including adults), found an incidence of 83.9%. Seizures represent a significant factor affecting the quality of life in children with MWS. Nevertheless, few large‐scale longitudinal studies have systematically characterized seizure types, EEG patterns, and long‐term outcomes in this population.

In our study, the age at seizure onset ranged from 1 month to 9 years 5 months (median: 25.5 months). The first seizure occurred before the age of 3 years in 75% of cases, and in three children, seizure onset was within the first year of life. One child presented during the neonatal period, underscoring the importance of considering neurological abnormalities in infants with suspected MWS. Mowat et al.^9^ noted that seizures occur most commonly during the second year of life. Similarly, Cordelli et al.^2^ reported a median onset age of 14.5 months in 22 children with MWS and epilepsy, with most seizures arising before age 5. Ivanovski et al.^8^ reported a mean onset age of 27.5 months. Our findings align closely with these reports.

In our study, fever‐triggered seizures were the initial manifestation in 18 of 31 children (58.1%), nonfebrile seizures in 10 (32.3%), and infection‐related seizures (without documented fever) in 3 (9.7%). Cordelli et al.^2^ concluded that 15 of 22 children (68%) experienced their first seizures during febrile episodes, whereas Ivanovski et al.^8^ reported fever‐induced seizures in 45% of patients. Collectively, these data suggest that heat sensitivity may represent a characteristic feature of MWS‐related epilepsy, possibly linked to *ZEB2*‐related neuronal excitability.

Focal seizures were recorded in all 31 patients (100%), confirming prior reports of their predominance in MWS (72.1% incidence).^8^ Notably, 16 children (51.6%) exhibited atypical focal semiology, including hyporesponsiveness, motionless staring, oral automatisms, and eyelid blinking without classical motor activity. Such subtle clinical features are easily missed or misinterpreted, especially in children with moderate‐to‐severe developmental delay.

Nearly half of the patients also displayed GI manifestations, abdominal distension, constipation, and vomiting. Among the 12 children with GI symptoms, nine had constipation or were diagnosed with congenital megacolon, suggesting a potential neurodevelopmental linkage between central and enteric nervous system dysfunction. Waheeda et al.^10^ showed that *ZEB2* plays a critical role in the development of enteric ganglion cells, whose impairment can lead to Hirschsprung disease and chronic constipation. Thus, the coexistence of neurological and GI symptoms in MWS supports the hypothesis of a brain–gut axis dysfunction.^10^, ^11^

Two children developed myoclonic seizures (at 3 years 8 months and 2 years 9 months, respectively); one patient initially presented with epileptic spasms meeting the criteria for infantile spasm syndrome. Another child developed atypical absence seizures at 7 years 4 months, followed by typical absence seizures at 7 years 10 months. This progression mirrors the age‐related evolution of absence seizures in MWS described by Cordelli et al.,^2^ who reported that atypical absences occurred in 59% of patients, generally after age 4, with none documented before age 6. Therefore, atypical absence seizures may be underrecognized in MWS, emphasizing the need for ongoing parental education and long‐term video EEG monitoring to ensure accurate identification and management.

EEGs from 31 children with MWS (87 recordings in total) demonstrated a clear age‐dependent pattern.^2^, ^12^ At seizure onset, the EEGs typically revealed slowing of the background rhythm, which gradually improved with age. Interictal discharges initially predominated in posterior regions and later evolved into multifocal or anterior‐dominant activity. A significant increase in epileptiform discharges was consistently observed during sleep. Additionally, ESES was detected in two children during follow‐up.

In contrast, a previous study on ESES in MWS‐associated epilepsy^3^ reported very early onset (2–5 years) in all five affected children and a markedly high spike–wave index (85%). Children with MWS who develop ESES often experience concurrent deterioration in cognition, behavior, and motor abilities. When abnormal discharges improve, either spontaneously or through clinical intervention, motor and behavioral functions may also recover. Therefore, if unexplained regression of cognitive or motor development occurs during the disease course, prompt EEG evaluation is warranted.

The *ZEB2* gene, localized at 2q22.3, plays a key role in the regulation of neurodevelopment^13^, ^14^ and remains the only causative gene identified for MWS to date.^4^, ^15^ In the present study, the SNVs/indels were predominantly nonsense and frameshift variants (25 cases, 24 different variants in total). Most variants were found in exon 8 (19 cases and 18 variants).

In a large study, Peter et al.^16^ analyzed *ZEB2* variants in 298 patients with MWS and reported frameshift variants in 45% (134 cases), nonsense variants in 38% (112 cases), and large segment deletions in 7% (21 cases). Additionally, exon 8 variants were the most frequent, accounting for 66% (198 cases). This high frequency likely reflects the relatively large size of exon 8 (~1.6 kb, 26% of the *ZEB2* coding sequence) rather than a true mutational hotspot. Kablan et al.^17^ similarly cautioned that exon‐specific frequencies must be interpreted with correction for exon length.

In this study, the recurrent variant c.2083C>T was identified in two unrelated children. This variant has been reported across multiple populations,^18^ with an overall frequency of approximately 11%,^16^ suggesting that c.2083C>T may represent a potential hotspot variant of *ZEB2*.

Three children carrying variants within the homeodomain (HD) region showed poor seizure control and persistent convulsive status epilepticus; two of these harbored c.2083C>T (p.Arg695*) and c.2002delG (p.Glu668Serfs*8). Among the four most common *ZEB2* variants, c.2083C>T is recurrently implicated in epilepsy. A literature review^19^, ^20^, ^21^, ^22^, ^23^, ^24^, ^25^, ^26^, ^27^, ^28^, ^29^, ^30^, ^31^ identified 22 reported children with this variant, 18 of whom experienced seizures, including one case of refractory status epilepticus.^28^ However, this preliminary genotype–phenotype correlation is constrained by the small sample size and possible ascertainment bias. Prospective, multicenter studies comparing HD versus non‐HD variants are warranted.

Previous studies^8^, ^32^ have shown that *ZEB2*‐related CNVs in MWS range from hundreds of kilobases to 16.7 megabases, and smaller deletions affecting single exons have also been reported. Cerruti et al.^24^ and Ishihara et al.^24^, ^33^ suggested that patients with large deletions exhibit more severe phenotypes. In this study, the four CNVs identified ranged in size from 136.01 kb to 12.16 Mb; however, no correlation was observed between deletion size and clinical severity. This lack of association likely reflects both the small sample size and potential position‐specific effects (e.g., haploinsufficiency of regulatory elements) that may override size‐dependent severity. Larger, systematically ascertained cohorts will be necessary to verify this observation.

The prognosis of seizures in children with MWS varies widely across the literature. Among the children with epilepsy in this cohort, 16 (53%) achieved seizure freedom for more than 6 months at the last follow‐up, indicating a generally favorable short‐term outcome. However, because the overall age of our participants was relatively young, continued long‐term follow‐up is required to assess sustained seizure control over time.

Ivanovski et al.^8^ reported that ASMs were effective in 74.1% of both children and adults with MWS, while Cordelli et al.^4^ reported a seizure‐free rate of 45% in pediatric patients despite an average use of three ASMs per case. Mowat et al.^9^ noted that seizures often become more controllable with age, suggesting that variability in reported outcomes may relate to differences in cohort age composition.

Regarding pharmacologic management, VPA has long been regarded as a first‐line ASM for MWS‐related epilepsy because of its efficacy in controlling both focal and atypical absence seizures.^2^ Consistent with these findings, VPA demonstrated good therapeutic efficacy in this cohort. LEV also showed favorable treatment effects: two children achieved seizure control for more than 2 years with LEV monotherapy, and five others maintained control for 1 to 2 years with LEV alone or in combination therapy. Of the 19 children receiving LEV, either as monotherapy or adjunctive therapy, 11 (57.9%) achieved seizure control lasting more than 6 months. In contrast, nine of 18 children (50.0%) treated with VPA achieved a similar duration of seizure control, suggesting that LEV may have comparable or even superior efficacy to VPA for MWS‐related epilepsy.

Two refractory cases demonstrated promising responses to newer ASMs: One child achieved 23 months of seizure freedom after the addition of PER to VPA and LEV, and another achieved 10 months of seizure freedom following the introduction of ZNS to a VPA and oxcarbazepine (OXC) regimen. These findings suggest that PER and ZNS may offer additional therapeutic benefits in the management of difficult‐to‐control MWS‐related seizures, although confirmation in larger cohorts is required.

This study summarized the clinical and genetic characteristics of 31 children with MWS carrying *ZEB2* variants, with a particular focus on seizure presentation and treatment. Seizures are common in MWS, frequently triggered by fever, and typically present before age 3 years, with focal seizures being the most prevalent. However, some children display atypical seizure manifestations that are subtle and easily overlooked. In addition to focal seizures, epileptic spasms, myoclonic seizures, and atypical absence seizures can occur. Convulsive status epilepticus is relatively frequent and warrants early recognition and aggressive management to prevent irreversible brain damage.

EEG abnormalities in MWS show age‐dependent evolution, and some children develop ESES over time. LEV and VPA appear to be the most effective and well‐tolerated ASMs for MWS‐related epilepsy, while PER and ZNS may serve as promising adjunctive therapies. The recurrent *ZEB2* variant c.2083C>T represents a potential hotspot mutation, and children carrying variants at this locus or within the HD may exhibit a genotype–phenotype correlation characterized by a higher seizure burden and greater treatment resistance. Further multicenter, longitudinal studies are needed to validate these findings and elucidate the molecular mechanisms underlying MWS‐associated epileptogenesis.

## AUTHOR CONTRIBUTIONS

Yi Ju: Resources; investigation; data curation; formal analysis; writing – original draft. Tao‐yun Ji: Resources; conceptualization; supervision; project administration; funding acquisition; writing – review and editing. All authors read and approved the final manuscript.

## FUNDING INFORMATION

This work is supported by the National High Level Hospital Clinical Research Funding (Multi‐center Clinical Research Project of Peking University First Hospital) (2022CR60).

## CONFLICT OF INTEREST STATEMENT

The author(s) declared no potential conflicts of interest with respect to the research, authorship, and/or publication of this article.

## PATIENT CONSENT

Written informed consent was obtained from all participating children or their legal guardians prior to enrollment in the study.

5

**Test yourself**

1. Which gene is responsible for Mowat–Wilson syndrome?
  - FBN1
  - ZEB2 (also known as ZFHX1B)
  - CFTR
  - TP53
2. What is the most frequently mentioned type of mutation in the article?
  - Missense mutation
  - Nonsense mutation
  - Frameshift mutation
  - Duplication mutation
3. Which type of seizure was most common among the 31 children?
  - Generalized tonic‐clonic
  - Focal seizures
  - Absence seizures
  - Myoclonic seizures
4. Which anti‐seizure medication(s) were highlighted as particularly effective in this study
  - Levetiracetam (LEV) only
  - Valproic acid (VPA) only
  - Both LEV and VPA
  - Zonisamide (ZNS)

*Answers may be found in the* *Supporting information*.

## Supporting information


Data S1

## Data Availability

The data sets generated and analyzed during the current study are available from the corresponding author upon reasonable request.
