# Supplementary material for: Electro‐clinical features of Mowat–Wilson syndrome: A retrospective study of 31 children in mainland China
Source: Epileptic Disord. 2025 Dec 27;28(2):344–58. doi: 10.1002/epd2.70149 (PMC13084205; doi:10.1002/epd2.70149)
Supplement: Supplementary file 1 — Data S1 [file EPD2-28-344-s001.docx]

Answers

1 B

2 B

3 B

4 C
